# A Three‐Domain Personality Analysis of a Mentally Tough Athlete

**DOI:** 10.1002/per.2129

**Published:** 2017-10-11

**Authors:** Tristan J. Coulter, Clifford J. Mallett, Jefferson A. Singer

**Affiliations:** ^1^ School of Exercise and Nutrition Sciences Queensland University of Technology Kelvin Grove Queensland Australia; ^2^ School of Human Movement and Nutrition Sciences The University of Queensland Brisbane Queensland Australia; ^3^ Psychology Department Connecticut College New London CT United States

**Keywords:** dispositional traits, characteristic adaptations, narrative identity, mental toughness, case study

## Abstract

The current study adopted McAdams' multilayer framework as the basis to develop a psychological portrait of an elite athlete who was identified as being particularly ‘mentally tough’. The aim was to use this single case as an exemplar to demonstrate the utility of McAdams' framework for understanding the complexity of sport performers across three domains of personality: dispositional traits, characteristic adaptations, and narrative identity. We operationalised these domains through the development of specific research questions and, subsequently, the collection and integration of the participant's Big Five traits, personal strivings, coping strategies, and response to a life story interview. The results offered a comprehensive insight into the nature of one athlete's personality that, in turn, informed conceptual perspectives of mental toughness in sport psychology literature and qualitatively supported emerging evidence of the validity of a three‐layer framework in personality psychology. Specifically, the study's design showed how a holistic approach to personality analysis can lead to a more complete psychological representation of competitors in sport, and people generally. It demonstrated how motivational, sociocultural, and meaning‐making aspects of personality can complement a trait profile to achieving a satisfying assessment of the whole person. Copyright © 2017 European Association of Personality Psychology

A recent review of the talent identification and development literature in sport suggests that personality is of high direct relevance to athletic success, regardless of one's ability (Rees et al., 2016). To understand this relevance more clearly, scholars have explored how personality impacts people's behaviours and experiences in sport. For instance, they have investigated how personality affects people's participation and engagement in this domain (Allen, Greenlees, & Jones, 2013) and their abilities to perform under pressure (Mosley & Laborde, 2016). In these research studies, scholars have consistently defined personality in sport in terms of (stable) traits (e.g. optimism and perfectionism). However, contemporary perspectives of personality support a more differentiated view of people's complexity, which includes, but also extends beyond, the trait domain (e.g. Dunlop, 2015; McAdams, 2013; Singer, 2005). For example, McAdams (1995, 2013) and colleagues (e.g. McAdams & Cox, 2010; McAdams & Pals, 2006) developed a whole person framework that integrates three different viewpoints: dispositional traits (the self as a social actor), characteristic adaptations (the self as a motivated agent), and narrative identity (the self as an autobiographical author). Recently, Coulter, Mallett, Singer, and Gucciardi (2016) have argued the potential of McAdams' integrated framework to advance personality research in sport beyond traits. Sport is not a common domain in which to find an examination of McAdams' (1995, 2013) framework. Hence, this context provides an alternative setting to the norm (e.g. political, clinical; McAdams, 2010; Singer, 2005; Wiggins, 2003) in which to gauge the general value of a three‐domain approach to personality analysis.

In the current study, we attempted to further clarify the utility of McAdams' framework for understanding personality in sport through a deep and integrated profile analysis of a single athlete. We also demonstrated the framework's potential to foster greater knowledge about psychological constructs relevant to sport and, in particular, to advance an understanding of a popular, but contentious, topic in sport psychology—namely, mental toughness (MT).

## Mental toughness

At a psychological level, 1Referring to MT at a ‘psychological level’ recognises the current trend in MT literature that sees the concept studied at different levels of scientific analysis (e.g. behavioural, sociocultural, and psychological). MT represents the essential blend of personality characteristics that enables performers to excel in achievement‐based contexts (Bell, Hardy, & Beattie, 2013; Jones, Hanton, & Connaughton, 2007). It is a concept that has become increasingly prominent in sport and other domains (e.g. business, education; Gucciardi, Hanton, Gordon, Mallett, & Temby, 2015) where importance is placed on identifying and developing people who can regularly perform to high levels, despite incidents of challenge, pressure, or adversity. However, despite such prominent interest in MT in recent years, what is meant by MT—its definition and conceptualisation—remains a highly debated issue (Cowden & Meyer‐Weitz, 2015; Tibbert, Andersen, & Morris, 2015).

This debate mainly stems from the different ways in which scholars have construed and studied MT. For instance, some scholars have drawn on conventional psychological frameworks to base their perceptions of MT. Included in this group is the work of Clough, Earle, and Sewell (2002), who recognised comparisons between MT and the stress‐buffering concept of hardiness (Kobasa, 1979). Clough et al. suggested that MT is, in essence, an extension of hardiness and subsequently devised a trait‐based 4Cs model, defined as performing well in challenging situations, commitment (to one's own goals), control (emotional control and life control), and confidence (interpersonal confidence and confidence in ability). Other approaches included in this category are those by Harmison (2011) and Hardy, Bell, and Beattie (2014), both of whom used existing personality theories to conceptualise MT in sport. Drawing on Mischel and Shoda's (1995) personality framework, Harmison (2011) proposed that MT could be understood as a complex and relatively stable social–cognitive construct. Specifically, Harmison articulated MT through a set of cognitive–affective processing units (featuring encodings, goals, beliefs, affects, and regulatory skills) that interact with one another, and an athlete's environment, to generate mentally tough behaviours. In contrast, Hardy et al. took a neuropsychological approach and connected MT with biological (behavioural inhibition, behavioural activation, and flight–fight–freeze) systems. In a study conducted with elite cricketers, these scholars used Gray and McNaughton's (2000) revised reinforcement sensitivity theory to associate mentally tough behaviour (coping under stress) with reward and punishment sensitivities.

Other research groups have taken a more exploratory, bottom‐up approach to conceptualising MT, whereby athletes', coaches', and other people's (e.g., sport psychologists and parents) perceptions of the concept have been examined via qualitative methods. For example, in interviews with Olympic and world champion athletes, Jones et al. (2007) sought to identify the psychological qualities of the ideal mentally tough athlete. Their analysis revealed 30 psychological attributes deemed essential to being mentally tough in sport, which were clustered under four separate dimensions—attitude/mindset (e.g. self‐belief), training (e.g. pushing yourself to the limit), competition (e.g. staying focused), and post‐competition (e.g. handling failure). More recently, based on their own unpublished interviews and review of the literature, Gucciardi et al. (2015) hypothesised seven trait‐based indicators of MT, namely, generalised self‐efficacy, buoyancy, success mindset, optimistic style, context knowledge, emotional regulation, and attention regulation. In a recent phenomenological study involving 10 high‐altitude mountaineers, MT was linked with an assortment of higher‐order themes, including suppressing emotion, task‐focused coping, maintaining perspective, and a desire to continue (Swann, Crust, and Allen‐Collinson, 2016).

Together, these efforts have produced different models of MT (e.g. Clough et al., 2002; Gucciardi, Gordon, & Dimmock, 2008; Jones et al., 2007). They have also resulted in the development of many inventories (Table 1). Overall, while some consistencies lie across the various conceptualisations of MT (e.g. it features attributes such as confidence, perseverance, and emotional control; Jackman, Swann, & Crust, 2016), the present image is that of a multi‐pronged concept, underlined by a range of competing assumptions.

**Table 1:** Psychological attributes used to conceptualise and measure mental toughness (MT)

Inventory	Dimensions/subscales
Psychological Performance Inventory (Loehr, 1986)	Self‐confidence, attention control, negative energy, motivation, attitude control, positive energy, visual and imagery control
MT Questionnaire‐48 (Clough et al., 2002)	Commitment, emotional control, life control, challenge, confidence in abilities, interpersonal confidence
MT Inventory (Middleton, Marsh, Martin, Richards, & Perry, 2005)	Mental self‐concept, potential, self‐efficacy, task familiarisation, personal bests, value, goal commitment, task focus, perseverance, positive comparison, stress minimisation, positivity
Psychological Performance Inventory‐Alternative (Golby, Sheard, & van Wersch, 2007)	Determination, self‐belief, positive cognition, visualisation
Mental, Emotional, and Bodily Toughness Inventory (Mack & Ragan, 2008)	Optimal performance state, empowering emotions, coping, well‐prepared, acting tough, flexibility, responsiveness, strength, resiliency
Australian Football MT Inventory (Gucciardi, Gordon, & Dimmock, 2009)	Thrive through challenge, sport awareness, tough attitude, desire success
Cricket MT Inventory (Gucciardi & Gordon, 2009)	Affective intelligence, attentional control, resilience, self‐belief, desire to achieve
Sport MT Questionnaire (Sheard, Golby, & van Wersch, 2009)	Confidence, constancy, control
MT Scale (Madrigal, Hamill, & Gill, 2013)	Using long‐term goals as a source of motivation, controlling the environment, pushing yourself to the limit, handling pressure, belief, regulating performance, staying focused, awareness and control of thoughts and feelings
MT Higher‐order Constructs (Guillén & Laborde, 2014)	Hope, optimism, perseverance, resilience
Academic MT Inventory (Amato‐Henderson, Slade, & Kemppainen, 2014)	Positive cognition, drive/determination, visualisation, impression management
MT Index (Gucciardi et al., 2015)	Generalised self‐efficacy, buoyancy, success mindset, optimistic style, context knowledge, emotion regulation, attention regulation
MT Psychological Skills Profile (Smith, Wolfe‐Clark, & Bryan, 2016)	Negative mindset, positive mindset, confidence, achievement, health behaviours

## McAdams' integrated framework and mental toughness

To address some of the conceptual ambiguity surrounding MT, one can be guided by McAdams' (1995, 2013) understanding of personality. McAdams proposed that personality can be described across three broad domains, or layers, that together yield a comprehensive profile of psychological individuality. The first layer encompasses dispositional traits, which account for the broad consistencies in people's behaviour, thought, and feeling across situations and over time. The second layer brings together a wide variety of characteristic adaptations that specify typical goals and values, and other aspects of personality, expressed in time, place, and social role. The third layer—narrative identity—involves the individual's integrative life story, reflecting the psychosocial construction of a personal identity and a framework for deriving meaning, unity, and purpose in life.

McAdams' framework enables scholars to integrate these differences under a single overarching structure and, thus, provides a basis for greater coherence in the MT literature. For example, different types of constructs have been used to describe MT. In some cases, these constructs have the properties of dispositional traits (e.g., Clough et al., 2002; Guillén & Laborde, 2014; Hardy et al., 2014), while in others they stem from different domains of personality (e.g. Harmison, 2011). McAdams' framework also offers a path to clarifying the nature–nurture debate in MT research. This debate focuses on the issue of whether MT should be treated as something dynamic (e.g. Bell et al., 2013; Connaughton, Wadey, Hanton, & Jones, 2008) or a more stable, trait‐like feature of personality (e.g. Clough et al., 2002; Guillén & Laborde, 2014; Hardy et al., 2014). An appealing feature of McAdams' framework is its ability to account for personality's continuity and change (McAdams & Olson, 2010). Hence, conceptualising MT across the three layers invites the prospect that it can encompass *both* stable and dynamic properties (i.e., traits, goals, values, and identities).

However, perhaps the most important use of McAdams' framework is the potential it bears for studying MT at a more comprehensive level of personality. Specifically, there has been a tendency for MT to be examined in a manner both arbitrary and overly narrow. For example, scholars have investigated the most important attributes of mentally tough performers but provided little guidance or ontological regard for making psychological sense of these individuals (e.g., Bull, Shambrook, James, & Brooks, 2005; Coulter, Mallett, & Gucciardi, 2010; Gucciardi et al., 2015; Jones et al., 2007; Jones, Hanton, & Connaughton, 2002). They have also tended to locate their research within one domain of personality (e.g., traits; Clough et al., 2002; Hardy et al., 2014), but with no clear rationale for doing so. McAdams' (1995, 2013) three‐domain framework could be used as a guide for developing more integrated questions about the personality of mentally tough competitors. The following are examples:

1. What *type* of people are mentally tough athletes?
2. What do mentally tough athletes *want and value* in sport, and how do they *function and adjust* to the challenges in this domain, in *context*, *time*, and *role*?
3. What gives mentally tough athletes' lives in time a *sense of meaning and purpose*?

These questions imply there are different aspects to people's personalities that contribute to a fuller account of their MT. In particular, they propose that people's traits, goals, regulation, and personal narratives play a role in explaining why some competitors might perform well under stress in sport (Hardy et al., 2014) or are more consistent than others in staying confident, determined, and focused (Jones et al., 2002).

## The Current Study

To illustrate the value of the McAdams framework for sharpening a conceptual understanding of MT, we have chosen to demonstrate its utility in an analysis of a case study of a high‐level professional athlete. The decision to study the personality of a single individual was fourfold. First, in‐depth case studies have the potential to supply important information for the development of new scientific insights and theoretical innovation (Flyvbjerg, 2011). Second, case studies allow for the detailed nuance that does justice to a multilayered construct (Thomas, 2011). Third, it answers scholars' calls to ground MT research in the real lives of athletes (Andersen, 2011; Tibbert et al., 2015). Finally, a single case allowed us to display the integrative nature of personality as a talking point for personality and sport psychologists to (i) conceptualise MT across multiple domains and (ii) gain greater knowledge and understanding of competitors in sport that includes, but also goes beyond, dispositional traits. In particular, it allows us to locate MT in contexts that include both cultural influence and questions of self‐understanding and meaning‐making.

In profiling the personality of someone identified as mentally tough, we were concerned with selecting constructs that conceptually articulated McAdams' framework, reflected the above‐stated research questions, and showed the potential value of understanding a mentally tough performer from a whole person perspective (Coulter et al., 2016). With these criteria in mind, four personality constructs were chosen for the current study. For the first layer, the Big Five taxonomy (Costa & McCrae, 1992) served as the basis for charting the broad *structure* of the identified athlete's personality. The Big Five is the most popular method for capturing dispositional traits and gives an insight to how people typically think, feel, and behave. For the next layer, characteristic adaptations detail people's typical goals and motives, and how they respond to stresses and vulnerabilities in time, role, and context. To explore the case study's *motivational agenda* in sport, Emmons' (2003) personal strivings measure helps to reveal individuals' recurrent goals tied to particular situations, roles, and time periods. To examine how the participant has *managed current stressful episodes* in sport, situational forms of coping were collected using Carver, Scheier, and Weintraub's (1989) coping orientation scale. For the third layer, we used a life story interview to examine the participant's *narrative identity*. By examining certain features of the life story (e.g. prototypical scripts, the meanings attached to critical life events; McAdams, 1988; Singer, Blagov, Berry, & Oost, 2013; Tomkins, 1979), we aimed to convey how this individual makes sense of his own life and what this means for understanding his investment in and commitment to sport.

Our preference for a person‐based psychology approach underpinned the methodology (Allport, 1937). This position backs the ‘middle way’ of personality psychology, which captures an understanding of personality that does not privilege either a biological or cultural determinism but acknowledges the synthetic contribution of both biology and culture (Singer, 2005). Underlying this assumption is the view that every person is in certain respects like all other persons, like some other persons, and like no other person (Kluckhohn & Murray, 1953). That is, people can be understood relative to normative means, but equally, through their unique phenomenological experiences, which together can form an integrative and distinctive whole. Hence, person‐based psychologists endorse the view that people are best understood through a combination of quantitative, qualitative, and interpretive methods (Singer, 2005). Embracing this complexity, mixed methods allow for a more complete and triangulated view of a studied phenomenon (Sparkes, 2015). They also promote a more complex and authentic assessment of reality (Mason, 2006), which matches our aim of merging disparate personality constructs (traits, characteristic adaptations, and narrative identity) into an integrated and person‐based analysis of an identified mentally tough athlete.

## Methodology

### Participant

A basic task in conducting any case study research is to define the unit of analysis (Yin, 2012). Defining MT is a debated topic among different research groups (Table 1). Hence, using one (or any) MT inventory to classify a standout ‘mentally tough’ individual is a potentially ambiguous and questionable task. This approach also invites other confounding difficulties (e.g. self‐presentation bias; Cowden, 2017; Hardy et al., 2014; Lin, Mutz, Clough, & Papageorgiou, 2017). As such, to find a suitable case study, we took an emerging perspective that sees MT portrayed as a socially constructed label reflecting certain prized behaviours and subcultural ideals in sport (Andersen, 2011; Eubank, Nesti, & Littlewood, 2017; Tibbert et al., 2015). This view implies that to know a mentally tough individual in any given context is to primarily acknowledge that he or she is somebody with a reputation for consistently meeting, displaying, and conforming to social standards and imperatives linked to the concept. We elected to draw on conditional behaviours and values specifically derived from an in‐depth subcultural understanding of MT in the same Australian Football League (AFL) club (Coulter et al., 2016) to help select an identified mentally tough case.

To do so, we re‐contacted six participants from the earlier subcultural study, who voluntarily agreed to support us in this effort. This cohort included three senior players, who were all members of the player's leadership group and had between 6 and 10 years professional playing experience at the club. It also included three full‐time coaches (including two ex‐club players), who had been part of the club's coaching team between 2 and 12 years. Initially, all participants were reminded of the behaviours and values connected with MT at the football club. Briefly, these behaviours were categorised under two main themes—self‐sacrifice and unrelenting standards—and included such actions as prioritising football in one's life (e.g. above relationships and other desirable pursuits), withholding emotions or personal problems, being an obsessive trainer and task perfectionist, and putting one's ‘body on the line’ during games. Underpinning these behaviours were four club values linked to MT that stressed the following required standards: a desire for constant improvement, a team‐first ethos, uncompromising effort, and the maintenance of an infallible image (Coulter et al., 2016). Against these subcultural and contextually nuanced indicators of MT, the six participants were asked to separately record and rank‐order the players they considered to be undeniably mentally tough in the current senior squad. No restrictions were placed on the number of players the participants could identify as being mentally tough. Upon completion of this task, all six participants nominated between four (*n* = 2) and five (*n* = 4) players into this category. From the ensuing list, representing eight players from a full squad of 42, a tailored scoring system was designed to determine the final rankings. This system was based on (i) the total number of occasions a player was nominated by the six participants and (ii) his position on each list (e.g. first and fourth), where scores ranging from 1 (ranked fifth) to 5 (ranked first) were assigned accordingly. Based on these criteria, from the players listed, one was identified as being mentally tough more so than others. This player scored 25 (out of a possible 30) in positional ranking scores, including being ranked first on three occasions. He was also the only player to appear on every list (i.e. a 100% nomination record). This individual was subsequently approached to participate in the current research. 2To provide a comparison, the second‐ and third‐placed players identified as being mentally tough respectively scored 16 (one first ranking) and 13 (no first rankings) out of 30 for positional ranking scores. They also correspondingly appeared in 80% and 66% of the lists.


An Australian male in his mid‐20s, called Wade (alias), voluntarily agreed to participate in the study. Wade has played Australian Rules football since late childhood and has represented State and All Australian sides during his youth (16–25 years). Wade was a high‐achieving schoolboy and is university educated. At the time of personality analysis, he had played in the AFL with the same club for almost a decade, including 2 years in a leadership role. He was also sidelined by injury and midway through a long‐term recovery programme.

### Measures

#### Big Five traits

The NEO Personality Inventory 3 (NEO‐PI‐3; McCrae & Costa, 2010) is a common measure of personality traits, which consists of 240 items measuring 30 traits that define the five‐factor model: neuroticism (N), extraversion (E), openness to experience (O), agreeableness (A), and conscientiousness (C). Items use a 5‐point Likert scale format, with responses ranging from *strongly disagree* to *strongly agree*. The NEO‐PI‐3 has two parallel forms: S for self‐reports and R for informant ratings. Both S and R forms were used in the current research (see procedure). The NEO‐PI‐3 has proven reliability and validity across a range of populations and cultures (McCrae, Harwood, & Kelly, 2011). In the current study, scores were interpreted for each facet and trait domain relative to adult male norms (McCrae & Costa, 2010) and categorised into a *very low* (at least two standard deviations below a mean score of 50), *low* (between one and two standard deviations below the mean), *average* (within one standard deviation of the mean), *high* (between one and two standard deviations above the mean), or *very high* (at least two standard deviations above the mean) range. NEO inventories have been used frequently in the sport context (Laborde, Breuer‐Weißborn, & Dosseville, 2013).

#### Personal strivings

To explore Wade's motivational agenda, Emmons' (2003) Personal Striving Assessment method was used. This measure was modified for context specificity by prompting Wade to list ‘the things I am typically trying to do in everyday behaviour in football’. Wade was asked to supply at least 10 personal strivings, on a page with space for 15. We provided Wade with examples such as ‘trying to support my teammates’ or ‘trying to avoid appearing incompetent’. We told Wade that he should not focus on how successful he might be in a particular striving but simply on how typically and frequently he pursued it. We also explained that the strivings should not be one‐time concerns but should be goal activities in which he engages on a regular basis. After writing down his strivings, Wade completed a Striving Assessment Scale, which involved rating each striving across four robust dimensions: *commitment* (the degree of commitment placed on a striving); *reward* (the degree of happiness related to each striving attainment); *challenge* (the effort required to pursue each striving, the probability of attainment in the future); and *causal attributions* (the internal or external reasons for pursuing each striving). Following Emmons' (2003) protocol, dimension ratings were based on two different rating scales. Degrees of commitment, happiness, and effort were rated on a 6‐point scale, ranging from 0 (e.g. *no happiness at all*) to 5 (e.g. *extreme happiness*). Probability of future attainment and causal attributions (extrinsic, introjected, identified, and intrinsic) were rated on a 10‐point scale, respectively, ranging from 0 (*0–9%*; *not at all because of this reason*) to 9 (*90–100%*; *completely because of this reason*).

#### Coping strategies

To identify the strategies Wade adopts to manage existing pressures in football, we used a time‐limited version of the Coping Orientation to Problems Experienced Inventory (COPE; Carver et al., 1989). The COPE is a 60‐item self‐report scale that assesses coping strategies clustered into three broad styles: problem‐focused coping (e.g. active coping), emotion‐focused coping (e.g. acceptance), and dysfunctional coping (e.g. denial). Wade reported on a 4‐point scale, ranging from 1 (*I don't do this at all*) to 4 (*I do this a great deal*), how often he used each coping strategy relative to a current, self‐proclaimed stressful episode in Australian football. Items are summed to produce scale scores, with higher scores reflecting greater use of a particular coping strategy. Total scores for each scale range between 4 and 16. Based around a mid‐range of 8–12, scores of 13 and above (i.e. 13–16) were treated as high, while those of 7 and below (i.e. 4–7) were considered low. The COPE has adequate validity, reliability (Carver et al., 1989), and factor structure (Litman, 2006) and has been used to measure people's coping style in response to stressors in sport (Kato, 2015).

#### Narrative identity

A life story interview was conducted to examine Wade's narrative identity. Using an adapted version of McAdams' (2008) life story protocol, this interview consisted of a combination of semi‐structured, open‐ended questions that encouraged Wade to identify important incidents and periods in his life and imagine these as cohering into an overall narrative. The interview included the main chapters in Wade's life; key scenes (e.g. high points, low points, and turning points); envisaged future chapters; main life challenges; personal ideologies and values; and football in the life story. Although the emphasis of the interview followed this broad plan, we anticipated that sport, and particularly football, would be a major focus of discussion. We also did not limit the interview to solely discussing Wade's life in sport. This decision was made to account for any life events occurring outside this domain that may have influenced or fuelled Wade's commitment in it. The interview requires 2–3 hours of time to complete, and despite its general structure, the tone of the interview is purposely conversational and participant led. It is noted that, while the interview aimed to obtain a story about Wade's life, he was being asked to do more than tell stories. Some questions were geared to pull story‐like responses (e.g. key scenes and life chapters). However, the focus was to elicit declarative statements about what this narrative currently means to Wade and identify prevailing scripts that form his identity.

### Procedure

After institutional ethical clearance had been granted, contact with Wade was initiated through his club's performance development manager. After the nature and requirements of the study were explained to Wade 3It is noted that, during the study, MT was not mentioned. Nor was Wade made aware that he had been selected for his MT. Instead, he was informed that the research aim was to understand personality in sport through a whole person approach. and informed consent was received, four meetings were arranged for Wade to complete the assessment protocol. Meetings lasted between 30 and 150 minutes and were conducted over a 3‐week period in the club's off season. To gain an accurate assessment of personality traits, it is advised that observer ratings be gathered when possible (Hofstee, 1994). During the first meeting, Wade was asked to nominate two individuals with extensive personal knowledge of him who would consider completing NEO‐PI‐3 informant forms (Form R) on his behalf—one in professional football (his AFL coach for the past 8 years) and another from a different domain (his partner of 11 years). The first author, an experienced sport psychologist, was responsible for all data collection. Following data analysis, two further meetings were arranged with Wade to discuss the personality report compiled by the research team. This report included assessment feedback and an integrated summary of results specific to the three research questions. The aim of these meetings was to collaborate with Wade and create an opportunity to promote new, co‐constructed insights towards developing a richer and fuller understanding of his personality (Sparkes & Smith, 2014; Tracy, 2010).

### Data analysis and integration

The NEO‐PI‐3 scores were analysed after all raw data (self‐report and informant) were entered into the NEO Software System and converted to *t* scores (Costa, McCrae, & PAR Staff, 2010). Scores were then explained using McCrae and Costa's (2010) trait descriptions, and personality‐style graphs were plotted. Cross‐observer correlations were also calculated, giving an indication of the relationship between informant and self‐report scores. The COPE inventory was scored following standard manual procedures and subsequently checked and confirmed by each member of the research team.

Thematic analysis of Wade's personal strivings was conducted across 12 categories of motivation, using definitional criteria provided by Emmons (2003). These categories include approach versus avoidance, agency versus communion, achievement, affiliation, and power. Notably, categories are not mutually exclusive. Thus, particular strivings could be coded into more than one category (or may not be codeable at all). The first two authors coded the strivings for motivational themes. Interrater agreement was determined via percentage comparison, where a rating exceeding 85% (Smith, 2000) was achieved.

The life story interview was audio‐recorded, transcribed, and analysed following procedures by Sparkes and Smith (2014). After immersing themselves with the data, the first two authors performed a thematic narrative analysis (Riessman, 2008) that focused on the key patterns identified in the life story, based on its content (i.e. *what* was said). This was followed by a structural analysis of narrative form, which concerns *how* stories are told, and the kind of narratives that are drawn on to scaffold and structure it (Riessman, 2008). Here, the authors were guided by McAdams' (1988) life story model of identity. This model specifies that narrative identity can be understood through the analysis of four major components: *ideological setting* (values or belief systems people use to suggest how the world around them operates), *imagoes* (archetypal characters that express idealised aspects of the self), *nuclear episodes* (significant self‐defining memories that shape people's lives), and *generativity scripts* (expectations about how one's story will end). The model also suggests that these life story components are woven together by two basic dimensions of theme and structure: *thematic lines* (the motivational currents that run through a given narrative) and *narrative complexity* (the degree of nuance, contradiction, and ambiguity built into the narrative). The use of McAdams' model helped to focus on the organisation and plot of Wade's life narrative and the main storylines running through his identity. After this initial phase, the third author, a specialist in narrative analysis, independently reviewed the interview transcript and initial findings. Through a process of triangulation among all authors, and the incorporation of member reflections reported by Wade during the collaborative process (Tracy, 2010), the key themes, plots, and narrative patterns were established.

To integrate the various forms of data, a three‐phase multilevel sequence was followed to generate an increasingly comprehensive and nuanced understanding of Wade (Anguera, Camerino, & Casteñer, 2012; Mason, 2006). Starting with the Big Five traits, we formed an emerging portrait of Wade's personality that proceeded to integrate data drawn from the subsequent layers of McAdams' framework. The rationale for this sequence was based on what McAdams' referred to as ‘the logic of person perception’ (McAdams & Manczak, 2011). This logic suggests there are different levels of understanding a person that range from macro (surface, comparative, dispositional; e.g. traits) to micro (deep, individualised, existential; e.g. goals and personal narratives) levels. At each phase of integration, we carefully considered what each layer and dataset suggested about the holistic profile of Wade's personality. To assist this process, we adhered to guidelines by Levak, Hogan, Beutler, and Song (2011), who give practical steps for integrating multiple datasets. One of these steps involved the process of dealing with conflicting material, which was useful for reaching a consensus regarding the main themes in Wade's personality. Another encouraged collaboration between the individual and his or her assessors, which was treated as a key step to compiling a fair and respectful report of Wade.

To encourage reflexivity and sincerity (Tracy, 2010), we also explored each other's roles in the research process. For example, we reflected on the specific relationship each author had with Wade (proximal vs. distal) and the impact this might have had on data interpretation. In particular, over the course of six meetings, the first author established a level of rapport with Wade that, if gone unchecked, had the potential to influence the main themes generated in the personality report. Establishing a good working relationship with participants is something to be encouraged to increase the chances of extracting quality data (Smith & Sparkes, 2016). However, it may also tap into a different domain of personality assessment (i.e. relational dynamics, Singer, 2005), which was not a goal of the current study. The role of the second and third authors was important, in this regard, both of whom had no contact with Wade throughout the duration of the research. These colleagues would assist the first author to reflect on his views about Wade, such as whether they were based on an interpretation of the actual available data or more so shaped by a relational impact during the consultations. Along similar lines, we also shared our unique perspectives regarding MT, including our biases for particular schools of thought on the topic. This collaborative step helped us gauge whether an overriding opinion of MT was taking precedence at any stage in the production of Wade's personality report. Any queries were resolved through ongoing reviews of the data and how such data linked back to our three original research questions. Overall, this continual shifting process reflects what Mason (2006) referred to as a *qualitatively driven approach* to mixed‐method explanation and the *creative dialogue* that can occur in collaborative mixed‐method research, where productive and innovative discussions help shape the quality and scope of the accounts developed.

### Trustworthiness

Criteria defined by Bryman, Becker, and Sempik (2008) were applied as a general means for promoting rigour for the mixed‐method design. These criteria suggest that mixed‐method studies should be relevant to the research questions and have appropriate rationales, transparent procedures, and integrated findings. Concerning the latter criterion, we followed O'Cathain, Murphy, and Nicholl's (2008) appraisal questions for assessing the integration process (e.g. ‘Is the type of integration stated?’). However, given the constructive and collaborative nature of our inquiry, its purposes, and the integrative procedure adopted that exemplifies our mixed‐method approach, additional quality measures were also deemed necessary (Sparkes, 2015). Hence, we offer the following bespoke criteria that are considered relevant for judging our mixed‐method design, for understanding mentally tough athletes, and the integrative findings that follow.

*Significant contribution:* Is the research relevant, timely, significant, and interesting?
*Expression of reality:* Does the work embody a sense of reality in understanding a competitor who is deemed to be mentally tough?
*Sincerity:* Is the study characterised by self‐reflexivity about subjective values, biases and inclinations of the researchers, and transparency about methods and challenges?
*Coherence:* Do the study's methods fit its stated goals and meaningfully integrate literature, research questions, findings, and interpretations with each other?

## Results and Discussion

### Wade's dispositional traits

**Figure 1:**
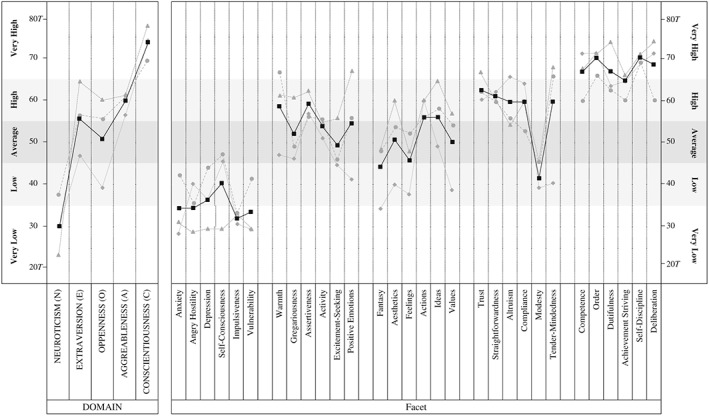
Wade's combined (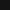), self‐rated (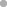), partner‐rated (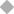), and coach‐rated (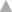) NEO Personality Inventory 3 profiles.

Wade's NEO‐PI‐3 trait profile is shown in Figure 1, representing both average and single (i.e. self and two observers) reports relative to adult male norms (McCrae & Costa, 2010). In what follows, we centre the interpretation of Wade's trait data on his combined profile score. Averaging NEO scores is common practice in reporting people's Big Five traits (McCrae et al., 2011). Likewise, high correlations were identified among the coefficients of profile agreement for each reporting dyad—Wade–coach (0.80), Wade–partner (0.66), and coach–partner (0.78) 4Further detailed information reporting the domain, facet, and personality style agreement scores across study dyads can be requested from the first author.—which add support for a combined profile assessment.

The most distinctive and uniform features of Wade's profile are his scores for C (*very high*) and N (*very low*). Wade places above the 99th percentile for C, meaning few other people are likely to be as motivated, strong willed, and persevering as he is, while his *very low* score for N (second percentile) indicates that he is a highly resilient and emotionally stable individual. Wade scored *high* for both E and A. Wade's *high* facet scores for warmth and assertiveness are the outstanding features for E. High scores for warmth suggest that Wade is affectionate and friendly and is somebody who usually experiences positive interpersonal encounters. His high assertiveness indicates that he is typically dominant and assertive in social settings—qualities reflective of people who become group leaders and actively seek out social rewards. A is a domain that measures people's interpersonal style. Wade's *high* (and *very high*) scores for five of the six facets of A indicate that he is a trusting, honest, and conforming individual, who has concern for others' welfare. However, Wade scored *low* for modesty, suggesting he is confident (even a little arrogant) when it comes to sharing his talents and achievements with others. Finally, the least distinctive feature of Wade's trait profile is his *average* score for openness to experience (O). Despite scoring average for O, three facets in this domain lie outside this normal range—fantasy (*low*), actions (*high*), and ideas (*high*). These scores suggest that Wade tends to be realistic in his judgements, enjoys new and different activities, and is open to new ways of solving problems.

We also considered the various paired combinations of his Big Five factor scores. McCrae and Costa (2010) identify 10 potential trait pairings, each denoting a particular ‘personality style’. Two personality styles were striking in Wade's profile: his style of *impulse control* and style of *character*. Wade's very low N and very high C placed him in the ‘directed’ quarter for impulse control (competitive orientation and tendency to be a ‘prototypical achiever’ who sets clear goals and works hard to attain high, socially meaningful standards of success; Piedmont, 1998). As for his style of character (high A and very high C), Wade was classified as an ‘effective altruist’ (i.e. someone who channels his efforts into serving others and is willing to take on and stick to difficult and thankless tasks until they are completed; McCrae & Costa, 2010).

#### Interpretation of Wade's dispositional profile

Using the NEO‐PI‐3, we have formed an outline of Wade's personality structure, which reflects the basis of his cognitive–affective and behavioural tendencies. People with similar traits to Wade are often described as optimistic and resilient, have high levels of psychological well‐being, and are conditioned for rewards and incentives (McAdams, 2015). They also tend to be hardy and emotionally stable under stress (Kaiseler, Polman, & Nicholls, 2012), selfless and sacrificing (Piedmont, 1998), and work hard to control instincts to attain success (McCrae & Costa, 2010). Wade's cardinal traits—typified by his style of impulse control and character—also combine to form a sketch of a person who (i) works tirelessly towards socially valued goals, (ii) can tolerate setbacks and unsatisfied needs, (iii) complies with the social order, and (iv) is willing to ‘work through the system’ to reach his aims (McCrae & Costa, 2010; Piedmont, 1998). Unsurprisingly, with these traits, Wade was identified as somebody deemed to be mentally tough (Coulter et al., 2016; Delaney, Goldman, King, & Nelson‐Gray, 2015; Horsburgh, Schermer, Veselka, & Vernon, 2009). At layer 1, then, Wade is someone with a highly controlled and focused personality (the *type* of person he is). However, traits are limited in what it can tell us about the whole person (McAdams, 1995, 2013). For instance, they cannot tell us what Wade *wants* from his involvement in Australian football, why he *chooses* to persist and not abandon his goals in this pursuit despite its demands, or the *strategies* he uses to deal with the pressures endured in this setting. To answer these sorts of questions, and to understand this mentally tough footballer more fully, we must go beyond his traits, and the Big Five, to the next layer of personality.

### Wade's characteristic adaptations

#### Personal strivings

Wade provided 12 personal strivings (Table 2). All of Wade's strivings were approach oriented, suggesting he is motivated by the prospect of experiencing positive events and possibilities in football, and a desire to thrive (and not merely survive) in this setting (Elliot & Sheldon, 1997). Nine of his strivings were also coded for agency. Hence, a basic feature of Wade's concerns in football is to get ahead of others (Hogan, 1982) and experience fulfilment through his accomplishments and mastery of the environment. Wade's remaining strivings were coded for agency–communion integration. This implies there is also a moral purpose inherent to Wade's motivations in football that reflect a desire to promote his own self‐interests by promoting the interests of others (Frimer, Walker, Dunlop, Lee, & Riches, 2011).

**Table 2:** Wade's Striving Assessment Scale

As a footballer, I typically try to …	Dimensions							
	Commitment	Reward	Ease/effort		Causal attributions			
	Commitment (0–5)	Happiness (0–5)	Effort (0–5)	Probability of future success (0–9)	Extrinsic (0–9)	Introjected (0–9)	Identified (0–9)	Intrinsic (0–9)
Make my life mean something†, §	5	4	2	8	4	2	8	7
Be as organised as I can†, §	4	5	3	8	2	0	8	7
Compete with others‡, §	4	3	4	8	3	3	7	7
Have a positive outlook†	4	2	1	8	2	0	9	8
Push my body to its limit†, §	4	4	5	8	2	0	7	6
Be a positive influence on those around me‡	4	4	5	7	5	3	8	6
Finish all tasks undertaken†, §	4	5	4	8	4	0	7	6
Learn something new†	3	3	3	7	3	0	8	7
Look for a purpose in what I do†	3	4	2	7	1	2	8	7
Be perfect in all I do†, §	3	4	4	7	2	0	6	6
Help others around me‡	3	3	3	7	4	3	8	4
(Be) logical in all thought processes†, §	3	3	2	8	2	2	7	5

† Strivings coded for agency.

‡ Strivings coded for agency–communion integration.

§ Strivings coded for achievement motivation.

Wade's strivings were found to manifest across five motivational themes. First, the degree of achievement content in Wade's strivings represents over half (seven; e.g. ‘push my body to its limit’) of those listed in Table 2. These strivings fit with both his high and very high scores on all six facets for C on the NEO‐PI‐3 and show the extent of his self‐demand and strong expectation for control, perfection, and closure in this context of his life. Second, Wade's strivings to help others, compete with others, and be a positive influence on those around him reflect his desire for power in football. Hence, asserting his influence or having an impact on others is a feature of what Wade wants. It also corresponds to his high level of assertiveness on the NEO‐PI‐3 and the current leadership role he assumes at his club. Third, two of Wade's strivings fit the motivational theme of personal growth. Having a positive outlook suggests Wade is someone who wants to keep things in perspective—a mark of his resilience and low score for N. His desire to learn new things shows his motivation to expand and develop himself in the game and resonates with this high score for action and ideas on the NEO‐PI‐3. Fourth, two of Wade's strivings were coded for spirituality, matching his high A and C on the NEO‐PI‐3 (Saroglou, 2010). Wade described that, through his football, he seeks to find purpose in his life and to make this life mean something. It was later revealed that these strivings reflect Wade's pledge to live a spiritual life, where football is a context that enables him to be a role model and achievement striver who fulfils the talents bestowed on him by a higher power. The final theme is self‐presentation. Wade was acutely aware of the social regard attached to some of his strivings. As a leading figure in the playing group, Wade stated the importance of portraying an image of a footballer who upholds the club ethos (e.g. ‘help others around me’) and behaves in ways that suggest a staunch commitment to achieve in the game (e.g. ‘be perfect in all I do’). This theme fits with Wade's high compliance and desire for recognition (high A and high E) from the NEO‐PI‐3. It also implies that what Wade wants in football partly reflects the values and norms held at his club, which has been a consistent feature in his development over the past decade.

In the Striving Assessment Scale (Table 2), Wade reported moderate to extreme commitment for all 12 strivings. He stated being very committed to six of these (e.g. ‘have a positive outlook’ and ‘compete with others’). However, the striving to ‘make my life mean something’ is his greatest possible commitment. Eleven of Wade's strivings received scores of extreme, very much, and much happiness upon attainment. The two that would give the most happiness are ‘be as organised as I can’ and ‘finish all tasks undertaken’. Two strivings require the highest level of effort to pursue for Wade—‘push my body to its limit’ and ‘be a positive influence on those around me’. Interestingly, ‘have a positive outlook’ was reported to demand the least amount of Wade's effort—this despite his existing injury status and projected long‐term recovery. Even for strivings that demand considerable effort and commitment from Wade, he predicts a very high probability (70–89%) that he will succeed in each striving in the future. In the final dimension, casual attributions, Wade rated all of his strivings highest in the ‘identified’ category. This implies that Wade pursues his goals primarily for self‐determined reasons and endorses them as important means by which he can succeed and stand out in this context (Deci & Ryan, 1985).

#### Coping strategies

Wade completed a time‐limited version of the COPE in relation to the most significant and self‐proclaimed source of stress currently consuming him in football. The COPE allowed us to focus more precisely on the strategies Wade uses to manage existing pressure in the game and fill in some of the detail of his general coping style derived from the NEO‐PI‐3. Wade stated that his injury and subsequent time out from the game was the most significant (and prolonged) source of stress at the time of his assessment. Dealing with and recovering from injury is a common form of psychological trauma in sport (Savage, Collins, & Cruickshank, 2016) and has been reported as the most common situation requiring MT in Australian football (Gucciardi et al., 2008).

We focused mainly on the most distinctive (i.e. very high/low) COPE scores as indicators of the strategies Wade uses to manage his injury‐related stress (Table 3). Under the category of problem‐focused coping, Wade reported engaging a great deal in active coping and planning strategies. This adaptive response (Carver et al., 1989) reflects Wade's very high facet scores for self‐discipline and deliberation on the NEO‐PI‐3, and his logical, organised, and controlled needs shown in his striving profile (Table 2). Under emotion‐focused coping, Wade reported relying heavily on his faith to support him in coping with his injury. Turning to religion can stimulate reinterpretations of negative events through a more spiritual lens and has been reported to play an important self‐regulatory role in the coping process (Pargament, 1997). He also stated that he employs positive reframing to view his injury in more helpful terms. Congruent with his positive outlook, Wade spoke about the potential benefits of his injury, suggesting this incidence has given him a sense of perspective and a renewed passion to play the game. This finding reinforces the mediating role positive reframing plays in explaining how injured athletes, who are high in trait hardiness, experience stress‐related growth (Salim, Wadey, & Diss, 2015).

**Table 3:** Wade's Coping Orientation to Problems Experienced Inventory scores

Coping style	Scale	Typified by	Total (4–16)
Problem focused	Active coping	Taking steps to eliminate the problem	14
	Planning	Thinking about dealing with the problem	13
	Suppression of competing activities	Focusing only on the problem	10
	Instrumental social support	Seeking advice from others	10
	Restraint coping	Waiting for the right moment to act	8
Emotion focused	Turning to religion	Using faith for support	14
	Positive reinterpretation	Reframing the stressor in positive terms	14
	Acceptance	Learning to accept the problem	10
	Emotional social support	Seeking sympathy from others	9
	Denial	Refusal to believe the problem is real	4
‘Less useful’/avoidant	Mental disengagement	Distracting self from thinking about the problem	8
	Focus on and venting emotions	Wanting to express feelings	7
	Behavioural disengagement	Giving up trying to deal with the problem	4
Other	Humour	Making light of the problem	7
	Substance use	Using alcohol or drugs to reduce distress	4

These problem‐focused and emotion‐focused strategies, and how they appear to be used by Wade, suggest he adaptively appraises his injury (Lazarus & Folkman, 1984), which is also reinforced by his low scores for less adaptive and avoidant coping responses. These findings mirror previous work that shows mentally tough athletes cope in similar ways (Kaiseler, Polman, & Nicholls, 2009; Nicholls, Polman, Levy, & Backhouse, 2008). That said, we found Wade's very low score of 4 for denial to be interesting, as it contrasted with later reports that suggest his role at the club at times affects the way that he copes. For example, the following quote depicts how Wade purposely portrays a positive image around coaches and teammates, which was interpreted as a form of denial in dealing with his current injury.

I have bad days, but I come in here [to the club] and I try to be as positive and upbeat as I can, when probably, deep down, I'm just like ‘I've had enough.’ Like, ‘I'm never going to [return to full fitness] again.’ That's why it's probably the hardest thing I've had to go through, because of those doubts I have.… But I try to put on a positive face for others. *I like people to know that I′m going okay*. I try to just deal with it, even though I know I'm feeling crappy. I like to encourage the guys and *be a leader for them* and, almost, how I'm feeling, you know, is secondary to that. (emphasis added)

Wade's account of putting on a ‘positive face’ to cope with injury, and how he likes others to know that he can manage his battles, implies a defensive and repressive side to his personality, where he seeks to present a positive appearance in his role at the club, independent of his actual levels of distress. 5On the assumption that Wade may be showing repressive and defensive tendencies in his coping, at the end of the study, we gained further ethical and informed consent to administer him the Weinberger Adjustment Inventory‐Short Form (WAI‐SF; Weinberger & Schwartz, 1990), which assesses an individual's social–emotional adjustment in the context of external constraints. Wade's scores on the WAI‐SF placed him borderline for ‘self‐assured’ and ‘repressive’ categories. Being ‘self‐assured’ suggests a well‐adjusted personality in coping with stress, while ‘repressors’ are known for being overly rational, giving the impression of self‐control, and working hard to maintain the belief that one is not prone to stress. Wade's score for repressive defensiveness (high) also indicated he is very keen to avoid negative evaluation and promote a positive façade. These scores reinforce how he reportedly regulates himself on the public stage at his club in coping with the demands of his current injury. Wade's willingness to engage in this form of coping (i) supports research suggesting that people high in MT have greater inhibitory control (Dewhurst, Anderson, Cotter, Crust, & Clough, 2012), (ii) reflects his desire to maintain a positive outlook and be a positive influence on others in football (Table 2), and (iii) bares the challenges associated with being or trying to be mentally tough, by virtue of his apparent need to conceal his vulnerabilities (Andersen, 2011). Arguably, such deliberate functioning results from a combination of personal and social factors that influence how Wade accepts his role at the club. Such factors include the synthesis of a compliant, dutiful, and incentivised individual (Figure 1) who has been coached for many years in a subculture that reportedly values selfless and infallible ideals (Coulter et al., 2016; Uphill & Hemmings, 2016).

#### Interpretation of Wade's characteristic adaptations

The analyses of Wade's personal strivings and coping data give a clearer understanding of him, not just as a ‘prototypical achiever’ and ‘effective altruist’, in the broadest sense, but of his personality in the context of Australian football. From his array of strivings, we have learned that Wade's long‐standing motivational agenda centres on a desire to get ahead and assert himself in this context. Wade's need for achievement is the main feature of his striving profile, reflecting his investment in agency through fulfilling rigid, self‐demanding goals. However, this need is not limited to egocentric desires. Wade's strivings suggest a genuine moral investment in helping support others, underlining his need for power in this context and the feeling he wants to lead by example. There is also a sense that Wade wants to discover his potential in the game. He is approach oriented, seeks to expand himself, and generally feels good when he satisfies his strivings. From Wade's profile, we can also see the confidence he exudes in football. Even for those strivings that require extreme levels of effort (i.e. pushing the physical limits and being a positive influence on others), he believes he can achieve his goals. He is highly committed to his strivings, but none more so than the desire to make his life ‘mean something’ through football. The spiritual subtext linked to this goal, and Wade's total commitment to it, suggest religion is interwoven into his psyche as a footballer. Reinforcing this view is the role his faith plays in helping him cope with current and significant levels of stress in the game. According to Emmons (2007), one of the basic functions of a religious belief system is that it provides an ultimate vision of *what people should be striving for in their lives* and *the strategies to meet those ends*. Spiritual strivings can also have a unique empowering function that energises people to persevere with their goals and invest time and effort towards their realisation, even under difficult circumstances (Pargament, 1997).

Furthermore, the analysis of Wade also shows the significance he places on self‐presentation in football. In line with his tendency to comply with social norms, coupled with a desire to achieve and get ahead, we now understand Wade as someone who adopts the ideals of his club and seeks to portray an image of a dutiful and hardy leader. This image is apparent in the approach Wade takes to deal with his existing long‐term injury. The strategies he uses to cope with this experience highlight the adaptive way that he deals with current high levels of stress, which reflect his propensity for emotional stability (Figure 1). However, one question his strong self‐presentation tendencies raise, which also reflects his very low scores for N on the NEO‐PI‐3, is how much he is invested in a repressive style that may not allow him to admit or acknowledge vulnerabilities. His quotation about the public face he shows in the clubhouse, compared to what he is feeling inside, does show recognition that he may have private concerns that he will not allow to come to the surface (Uphill & Hemmings, 2016). However, his capacity for this introspection mitigates against portraying him as caught in a repressive coping style that pushes negative affect out of awareness.

### Wade's narrative identity

Wade divided his life story into five chronological chapters. In Chapter 1, titled ‘Childhood Years’, he recalled his earliest boyhood memories growing up in a major Australian city with his family. He described this as a very enjoyable period of his life, which often revolved around the family's collective love for sport and competitive backyard games. In Chapter 2—‘High School Years’—Wade described making a ‘gut feeling’ decision at 12 years old to attend a Christian high school (versus joining the local State High like most of his friends). He recalled many fond memories of his time at school, including building strong relations with peers and teachers, and being instilled with the religious values and beliefs that he lives by today. Wade also defined himself as a high achiever. ‘I tried to make sure that I did well in everything’, he recalled. ‘If I wasn't in the top couple in everything I did, I'd be reasonably disappointed’.

In Chapter 3, ‘Realising and Chasing My Dreams’, Wade recollected the time he scaled the junior ranks playing Australian Rules football. By the time he was 13 years old, he said he knew that he wanted to play the game professionally, even remembering the specific moment when this goal became clear to him (see below). In this chapter, he recited a string of successes as an adolescent player and how he made various state and national age group teams, including several as captain. During these recollections, Wade underlined the preference he had for participating in team sports, especially the enjoyment and comfort he gets from working with others to achieve success. However, up to the age of 12, Wade was prohibited from participating in football, because of family church commitments. Despite describing his parents as ‘extremely supportive’ of the whole family, on this specific matter, Wade remembers having to strongly convince them to eventually let him play on Sundays.

Despite his feats as a junior hopeful, Chapter 4 (‘Draft Setback’) represented the time when Wade, at 17 years of age, experienced ‘the sick and hollow feelings’ of failing to get drafted into the AFL. He remembers being ‘extremely disappointed’, because he believed football was his destiny. ‘That was my dream and I thought that was what I was here to do’. However, not to be put off, Wade remembers quickly deciding to keep perspective and renew his commitment to playing in the AFL. He deferred university and got a job for a year, which he saw as significant to his eventual transition interstate to his current AFL club. ‘I grew up … [and] I look back on it now and think it was probably the best thing for me. It just helped teach me a lot of things and put a bit of drive into me as well’.

Up to the point of his current injury, Wade identified the final chapter of his life story as the time he has spent playing in the AFL (Chapter 5; ‘AFL Years’). He told how he got drafted the next year, as an 18‐year‐old rookie, and was then faced with the reality of life as a professional footballer. On the field, Wade played over 10 senior games in his first season and over 20 in his second. Early in his third season, however, he was dropped. ‘I fell out of favour with the Head Coach’, he said. ‘I'd just started going to uni part‐time and I'd just got married. The club were using these as excuses for why I wasn't performing well enough’. Wade was not selected to play for the rest of that season. However, despite this challenge, he recalled using his disappointment as a spur to get back into the team. ‘I got a lot fitter and a lot stronger than I ever was and I knew that all I needed was an opportunity to play’. That opportunity came early the following season, after which Wade played every game in the senior team. When asked about how Wade was able to turn things around, he said:

Part of it was that I think I could always play.… Also, I always had a feeling inside that it was what I was here to do. Since I was 13, I just always felt like it was my purpose to play AFL. I have always known that …. It's always been something inside.

Wade then recalled how he had won the club's Best and Fairest award 6Best and Fairest awards are common in Australian sport given to the player(s) judged to have the best performance over a season for a given sporting club. that year, receiving it again in two of the following three seasons (he was runner‐up in the year he did not win). During this time, Wade was also promoted into the club's leadership group and later awarded the club captaincy, which he has held for 2 years at the time of this analysis.

In addition to recalling his life story as a series of chapters, Wade identified nine key scenes and episodes that he judged to be highly significant to him (Table 4). These *nuclear episodes* are filled with meaning and emotion and include Wade's childhood recollection of playing backyard sport with his siblings and the moment when he was named club captain. A feature of the memories in Table 4 is the high degree of integrative meaning—the life or moral lessons—Wade assigns to them. For example, the incident of his brother's intended suicide reminded him of the importance friends and family in his life; being dropped in his third professional year was the catalyst, in Wade's mind, that led him to redefine his approach in football—‘*That* [event] drove me to improve’ (emphasis added). The integrative nature of Wade's memories, and the positive lessons attached to them, suggests he sees them as key episodes that have allowed him to achieve his most important goals to date (Erikson, 1958). The high number of integrative statements in his memories, and the high level of complexity in his storytelling, also points to a strong degree of adjustment and emotional maturity (Blagov & Singer, 2004).

**Table 4:** Key scenes in Wade's life story

Nuclear episode	Wade's reflection
High point: chosen to be captain of an Australian Football League (AFL) team	Wade shared how he had always harboured the ambition to be captain. He saw the importance of the event in terms that went beyond himself. ‘I'd like to think that I can influence people positively in this world. Like, I'm not just here so I can live for myself and live selfishly, but actually, so I can help as many people as I can and be a great influence on them.’
Low point: brother planned suicide attempt	Wade recalled feelings of helplessness during his early 20s when his brother had planned to commit suicide. With his brother's eventual recovery, the significance of the scene reminded Wade of how valuable his friends and family are in his life.
Turning point: decision at 12 to attend the Christian School	Wade remembers crying and begging his parents to let him attend the school. ‘I had this desire within me to go there. It was something internal. A quite voice inside that said, “This is the right place.” ’
Positive childhood memory: playing backyard sport with siblings	‘Just kicking the football out the front with my siblings. The enjoyment and being outside, competing, and that enjoyment from it.’
Negative childhood memory: notable for his inability to recall one	‘The majority of my childhood was very positive. I've been very lucky.’
Wisdom event: getting married at age 20	‘I suppose people look at it, especially at a footy club, and say, “What are you doing?” But I've got certain beliefs and values, and I'm willing to stick with them and follow through, and not just compromise them because it's not cool or against the grain.’
Religious, spiritual, or mystical experience: (i) a calling to play AFL football at 13 and (ii) the word of God at a time of uncertainty	Wade described two poignant spiritual memories: (i) at age 13 at Youth Group, Wade's Pastor discussed gaining a calling and he (Wade) realised that his was to play professional football. ‘God will give you the dreams and desires of your heart … and my desire was to play AFL and I just knew it. Like, at that time, it just felt sealed within me. It was what I was going to do.’ (ii) During Wade's third AFL season, when he had been injured and later dropped, Wade described how his Pastor ‘spoke a word over me and everything he said has come to pass.’ The significance of these experiences has given Wade the peace of mind he is playing football for a reason. ‘I'm here for a purpose, and I know I'm here, not just for me, but to [positively] influence others.’
Greatest life challenge: upholding integrity at AFL club	Wade talked about the inner conflict he experienced during his early professional years in attempting to balance the desire to feel accepted into the culture at his club without compromising his own values. ‘Standing up against that longing just to feel like one of the boys and not feeling ashamed because I wasn't doing what they were doing.’ Regarding the importance of the event, Wade said, ‘It stops you from compromising who you are—being someone who has integrity, who doesn't just go with whatever everyone else is doing. But making that conscious choice to say, “No, this is who I am and this is what I want to do and live by.” ’
Most significant event in football: being dropped	‘That drove me to improve and gave me the confidence that I could be a regular first team player.’ He believed he would not have been given the captaincy or got ‘my body to the point where it needed to be’ if it had not been for that period and setback.

We also note the redemptive sequences inherent to the way that Wade recounts many of his self‐defining memories. For example, at 12, Wade begs his parents to attend a Christian high school, to which he attributes the platform for his personal growth and spiritual development; at 17, Wade misses out on draft selection but is successful the following year; he gets dropped from the senior team but later wins two Best and Fairest honours and is awarded the club captaincy; his brother makes an eventual recovery from mental illness; Wade realises he is a man of integrity, despite experiencing strong doubts when attempting to fit into his football surroundings as a rookie. These sequences stand out as psychologically significant patterns in Wade's life story that reflect his tendency to describe meaningful scenes that change from bad to good. People who construct positive resolutions to difficult life experiences often report higher levels of resilience (Bonanno, 2004), well‐being (McAdams, Reynolds, Lewis, Patten, & Bowman, 2001), and emotional adjustment (Dumas, Lawford, Tieu, & Pratt, 2009). The continuity of Wade's redemptive memories also suggests he has developed a personal script (i.e. an internal schema; Singer et al., 2013; Tomkins, 1979) for resolving challenging events in his life. In a football context, this script filters his cognitive–affective processing represented by an emotion outcome sequence of *challenge–optimism–achievement*. In other words, when Wade experiences a setback (e.g. being dropped), after the initial negative affect, he is likely to respond with a sense of hope and renewed commitment that ends with a positive and rewarding outcome (e.g. reselection). At the conclusion of Wade's interview, he was asked to describe any major themes in his life story. He identified ‘coming through setbacks’ as a major thematic line in his personal narrative. His current injury might also have evoked past memories that underline this redemptive theme (Singer et al., 2013), connecting this episode with other difficult times in Wade's life that remind him of his ability to ‘dig deep’.

‘Being true to my beliefs’ was the second major theme Wade identified in his life story. When asked about the beliefs and values he lives by, Wade said:

I believe Jesus died on the cross for my sins and I believe I'll go to heaven. I'm here to love people and do life with them and, hopefully, through the example of my life, they will see Him in me.

This example translates into Wade's self‐proclaimed leadership style as club captain, which emphasises a genuine interest in supporting others, ‘not just using them to get where I want … but ensuring that they leave this place as better people’. This narrative backdrop (*ideological setting*) reinforces spiritual themes identified in the analysis of Wade's characteristic adaptations. But, at this third layer of personality, we have a much deeper sense of the standing Wade's faith plays in how he understands who he is, and critically, how this connects to his sporting goals. In particular, his quest to become an AFL player (‘my dream’), and to be successful in this pursuit, drives the story's plot. Interwoven with this primary goal, however, is Wade's firm belief that he has been guided by God to take this chosen path, reflecting a sacred mission, ‘sealed’ within him since he was 13 years old, that provides his identity with a clear and formidable sense of meaning and purpose—‘*This* is what I'm here to do’ (emphasis added). This spiritual conviction and imagined connection appear in various forms throughout his story (e.g. a spoken word and a quiet voice; Table 4) and act as both an inner guide and sanctuary as he faces his world.

The events and episodes Wade describes in his life story also suggest he views the outside world with a high degree of trust and certainty. He has developed an understanding of positive agency and considers himself an active agent able to achieve socially desirable outcomes (e.g. accolades) and be a positive influence on others (e.g. as captain). Furthermore, Wade identifies how ‘blessed and fortunate’ he has been in his life, conveyed through the opportunities (e.g. leadership roles) and life circumstances (e.g. being in a loving and supportive family) afforded to him. This sense of privilege, coupled with the peace of mind and guidance Wade draws from his faith, and overall assurance in his abilities, suggest he sees the world, and his future in it, as one full of optimism and promise.

Looking to the future, Wade foresaw himself as someone who can positively contribute to his community—‘using who I am and what I do for something good’. In football, he anticipates recovering fully from his current injury and leading his team to a Premiership win in the coming years. As Wade puts it, the personal accolades attained thus far in his playing career ‘mean something … and are better than half or three quarters of the players who ever played the game’, but they are not enough to satisfy him ‘without that win’. For Wade, the significance of winning a Premiership flag is twofold. First, it fulfils his boyhood dream of achieving ultimate success, and poignantly, the satisfaction of sharing that success with others—‘That feeling of doing it with 20‐odd other players and coach. That is why I took up the game’. Second, it symbolises Wade's personal legacy (*generativity script*) to redeem his club of, what he describes as, its ‘overlooked’ and ‘downtrodden’ reputation in the AFL. To elaborate, Wade spent some time during the interview describing how he had developed in a club that has generally perceived itself as ‘the underdog’ compared to its rivals. He discussed the various financial and franchise difficulties the club has had over recent years and his views about its unequal treatment by outsiders (e.g. the media). Wade told how developing in this environment over time has instilled a degree of ‘fight’ in him, and a motivation ‘to prove people [i.e. the sceptics] wrong’. He reported how winning a Premiership would help get the attention and respect the club deserves.

Together, these narratives resemble what Tomkins (1987) called a commitment script, emphasising the story of a protagonist who doggedly pursues a lifelong goal. The latter also depicts a redemptive theme of upward social mobility (McAdams, 2015), wherein a club that lacks the resources and attention of its competitors beats the odds to reach the highest honours. Research suggests that commitment scripts and stories of redemption are often told around a particular narrative structure (e.g. McAdams, 2015; McAdams, Diamond, de St. Aubin, & Mansfield, 1997), where the protagonist (i) experiences an early advantage or blessing, (ii) shows sensitivity to the suffering of others, (iii) establishes a clear personal ideology in adolescence—often linked to religion—that remains a source of unwavering conviction through the adult years, (iv) redeems bad events into good outcomes, (v) expresses strong motivations for both agency and communion, and (vi) sets optimistic and prosocial goals for the future. Wade's life story follows this format. The protagonist reports many advantages and blessings, especially in family, school, and sport (‘I've been very lucky’). He shows sensitivity to the suffering of others told through the tales of his brother's challenges and the alleged inequities experienced at his club. This sense of being privileged in life, while observing the suffering of others, affirms the protagonist's conviction that he has been ‘called’ to do good things and make a positive difference in the world (McAdams, 2015). This commitment and sense of destiny is bolstered by a clear and resounding religious belief system that remains steadfast over time. Seeing one's life in terms of redemptive sequences, furthermore, provides the protagonist the belief that persistence pays off and the hard work that goes into one's goals will eventually yield positive outcomes in the future (McAdams, 2015). His desire for power (recognition, self‐enhancement) is fulfilled in the service of communion (safeguarding the club, positively developing others). Finally, looking to the future, the protagonist sets forth goals and plans that aim to benefit both the self and the wider social world (e.g. ‘using who I am and what I do for something good’).

In the analysis of Wade's personal strivings, we learned that Wade's greatest commitment in football is to make his life ‘mean something’. We can now expand and connect this finding to Wade's moral obligation to deliver success to his club. Doing so leaves behind a positive legacy that satisfies both Wade's personal (needs for status and affiliation) and social (‘to prove people wrong’) goals. It provides him a psychological resource to persevere with the demands and setbacks endured in AFL football. It also helps sustain his conviction that the efforts and sacrifices put in today will ultimately be redeemed.

When Wade's narrative is reviewed, it becomes clear that the thematic lines in his life story reflect a blend of his needs for separation (agency) and relationship (communion; Bakan, 1966). This mix is exhibited in the four main characters, or *imagoes*, that define his life story. The first character, the Playful Child, is introduced to us at the very start and denotes an idealised characterisation of the safe, secure, and supportive surroundings of Wade's upbringing. The second protagonist is the Agentic Athlete. Much of Wade's life story is organised around his journey to become a professional AFL player and his efforts at the elite level. The Agentic Athlete represents Wade's goal to be a competent and recognised footballer, who has the desire to prove his worth through his performances and ability to stay true to this commitment, despite its challenges. The third imago, the Good Christian, personifies the most important value in Wade's life—his spiritual and caregiving obligation to support and nurture humanity. His personal myth is, in part, the story of a religious advocate who strives to lend himself to the betterment of others through generosity and example. The Good Christian also symbolises Wade's sense of belonging to the group and a desire to collectively share success with others.

Together, the three characters are idealised imagoes that personify the general agentic (Agentic Athlete) and communal (Playful Child and Good Christian) themes in Wade's life story. They also set up a prevailing dialectic in his narrative identity (Gregg, 1991), represented by internalised images of achievement and separation (i.e. power), on the one hand, and a desire to closely relate to and care for others (i.e. love), on the other hand. To resolve this tension, Wade projects a fourth imago into his life story, the Heroic Leader, which integrates these figurative opposites into a coherent and dynamic whole. The Heroic Leader is the most profound imago in Wade's life story, which, together with his own goals, is built around the idealised image of one of Wade's former captains, whom he described as the most important influence on him in the AFL. This main character typifies Wade's desire for prestige as a leading player at his club and a self‐personification of a respected role model who can influence and support others in positive ways. It also expresses an image of a moral and prosocial individual, who can relate to others and inspire them to follow his vision. Like Moses leading the Israelites out of Egypt, this vision reflects Wade's ambition to lead his club out of AFL obscurity to the heights of a Premiership win. 7The metaphor linking Wade's leadership goal with the biblical tale of Moses and the Exodus was shared with him during the collaborative debrief of his narrative analysis. We include it here because Wade agreed with the parallels being suggested. That is, a man of faith leading his men from injustice to the Promised Land. At its core, the Heroic Leader imago is a moral exemplar signalling Wade's motivation to promote his own self‐interests through promoting the interests of others (Frimer et al., 2011). This characterisation weaves agency and communion together within the narrative flow—‘I'm playing football for a reason … not just for me, but because I'm here to influence others’.

#### Interpretation of Wade's narrative identity

To summarise, Wade's narrative follows the ambitions of a boy who grows up to realise his dream of playing in the AFL. Entwined with this ambition are the events and circumstances that have convinced Wade of God's presence in guiding and supporting him in this pursuit. Despite experiencing various challenges and disappointments along the way, Wade's ability to muster up hope and commitment transform his setbacks into enhancement. Significant rewards and accolades follow, reinforcing the identity of a gifted and resilient individual with integrity. However, on their own, the rewards and accolades are not enough. As a self‐proclaimed leader and club captain, the story casts an image of Wade delivering a Premiership win. This victory reflects Wade's idealised commitment to a lasting contribution and the integration of a personal narrative emphasising his desire for power and redemption. It also expresses Wade's deep conviction that he has been ‘called upon’ or ‘chosen’ to be a positive influence in the world.

Consistent with our analysis at the first two layers of personality, Wade's life story contains themes of a goal‐directed agent who aims to give back to others in light of the fortunes that he has enjoyed. Reflecting the tale of a personal mission, Wade's narrative is a story about a religious individual's quest for achievement and legacy that plays out in a sporting context. It is this story that Wade carries around with him, implicit and evolving over time, which speaks of his narrative identity. With profound continuity and coherence, it sets him up, psychologically speaking, to endure the challenges and duties that accompany the hard task of succeeding and surviving in professional football. Put simply, it is the key to his MT.

For Wade, the hardest task, at present, is tolerating the stress and arduous hours of rehabilitation associated with his injury, all the while maintaining a certain façade, which he feels is necessary, in his role as a leading figure at the club. What assurances are there to suggest that Wade can sustain the commitment and strength of mind to endure this latest challenge? First, we might suggest that he possesses a particular trait profile (e.g., very high C, very low N) that is favourable in this regard (Figure 1). Similarly, his motivational agenda (e.g. his desire to get ahead, to be a master of his environment), and the strategies he uses to cope with his injury, may support his efforts and resolve to get through (Tables 1 and 2). In addition to these internal resources, however, is a personal narrative that provides Wade's life in time with a clear sense of meaning, unity, and purpose. Layered over his traits, goals, and coping strategies is an internalised story carrying themes of achievement, faith, and legacy that picks him up when things get bad and remind him that the sacrifices that football demands of him today will be worth it in the end. The structure of this narrative is also indicative of, what Singer (2001) calls, a highly functioning or ‘healthy’ individual. Singer suggests that optimal well‐being is reflected in stories that (i) contain a balanced sense of agency and communion, (ii) are infused with an overall setting of hope and possibility, (iii) find positive meaning out of disappointments, (iv) tolerate ambiguity and contradiction, and (v) express a conviction that one can offer something of value to the world. Alongside Wade's dispositional traits and characteristic adaptations, then, is an upbeat and adaptive story, which is filled with drive and redemptive scripts and suggests a degree of confidence and hope that Wade can reach a positive outcome to this latest setback.

The analysis of personality via the life story interview also brings to attention the role of culture in the construction of Wade's narrative identity. From previous work (Coulter et al., 2016), we have some understanding of the cultural context in which Wade is embedded in football. For example, we know he plays for a club that historically sees itself as ‘disadvantaged’ compared to its rivals (p. 104). We also know that it ratifies MT as a term expressing salient subcultural values and ideals, such as being selfless, infallible, and uncompromising. Similar themes, or ‘master narratives’ (Hammack, 2008), appear in Wade's life story (e.g. ‘to fight for what you wanted’, ‘to succeed against the odds’). These themes are prominent in Wade's recollection of his early years in professional football—‘something that was impressed upon you, even without you realising it’—as well as in the connections he makes between these memories and his existing narrative identity. A review of Wade's life story suggests he has assumed and personalised these prevailing master narratives at his club, subsequently shaping his identity as a result of his unique social circumstances in the professional game.

Furthermore, McAdams (2015) states that varying kinds of narrative identities make sense in different cultural contexts (also Hammack, 2008). In a context that exhibits ‘the underdog’ mentality, Wade's story of a hardy leader, who commits himself to redeem his club's identity, probably makes good sense to the many people (teammates, coaches, fans, the board, etc.) connected with this particular organisation. Given that Wade is the type of person who is inspired by social recognition (high E and high A; Figure 1) and strives to make his life ‘mean something’ through football (Table 2), it is perhaps understandable he has fashioned this particular story for himself, which lines up his own unique experiences with what his existing football (indeed, family and religious) culture indicates is a meaningful life. Overall, Wade's life story displays a strong degree of interdependency between the person and context, reinforcing McAdams' assumptions of the relationship between culture and personality. On the one hand, Wade's club has provided him with the opportunity to fulfil his destiny, which he initially imagined as a 13‐year‐old boy. On the other hand, there is a feeling that the club needs him as much as he needs it. Together, these perspectives give further clarity and meaning to Wade's identity in football and cement his own view that he has been ‘chosen’ to redeem his club's hard‐up status.

‘We are the stories that we tell’, says McAdams (2006, p. 3). Or more precisely, our stories are an important part of our personality, along with other parts, like dispositional traits, goals, and coping strategies (McAdams & Manczak, 2015). What may differentiate Wade's personality from others is the way in which he has configured a personal narrative that gives unity and purpose to his existence. In other words, through the memories, stories, and images of his life, *Wade knows himself* as a person who can achieve socially desirable goals, be of service to others, and turn setbacks into gains. Hence, whilst capturing dispositional traits and characteristic adaptations are important for understanding Wade's MT, a much clearer and more satisfying portrait is achieved if we also know his story. It is about understanding how Wade organises the memories and events of his life into a meaningful narrative that goes some way to explaining his commitment to achieve in football and why he sustains this commitment despite the pressures and setbacks incurred throughout his involvement in the game.

## General Discussion

This study takes an alternative approach to understanding MT, calling for a deeper and more complex representation of people identified as being mentally tough. Specifically, we analysed the personality of a single athlete using McAdams' (1995, 2013) integrative framework, marking an effort to bridge the gap between contemporary perspectives in personality psychology and those in sport that tend to characterise personality through a single trait domain (e.g. Allen et al., 2013; Mosley & Laborde, 2016; Roberts & Woodman, 2016).

### Contributions to mental toughness literature

This work advances the existing conceptual debate in the MT area and contributes to the literature in four key ways. First, the three research questions, reflecting each layer of McAdams' model, provided a greater degree of structure and coherence that has been missing in previous MT inquiries. These questions focus on examining core dimensions of psychological individuality and emphasise relatively stable aspects of personality that include, but also go beyond, trait descriptors. Importantly, they provide a platform and focus for scholars to comprehensively assess and ponder the personalities of mentally tough performers. As a result, the development of such research questions offers new opportunities in this space, especially in terms of study design (more on this issue below). In the past, scholars have often relied solely on the self‐reports of participants to determine what is important to know about the psychology of MT. Although there is some rigour to this approach, the current study extends this effort by providing an overarching guide for designing future conceptually focused research that accounts for key aspects of the whole person. Crucially, it illustrates that the underlying motivations and meaning structures behind individuals perceived as mentally tough are likely to show variation that grow out of both personal experience and sociocultural influence. Wade's personality is linked to a powerful moral and religious substrate that defines his life's purpose. Another individual with a similar surface presentation of MT may have an underlying meaning structure linked to narcissistic injury and a selfish desire for glory (e.g. Onley, Veselka, Schermer, & Vernon, 2013). The three‐level analysis enables a deeper and more differentiated examination of MT and, thus, warrants the prospect that mentally tough individuals are driven by different internal forces.

A second related contribution is the use of a case study approach to advance conceptual perspectives in MT research. This case study celebrates the particular over theoretical abstraction and captures personality in the context of the participant's own social circumstances. In this sense, we have presented a multidomain portrayal of a competitor that emphasises an integrated whole, rather than the reduction of a life to the sum of scalable individual differences, which is the norm in current MT research. It is our attempt to move closer towards a more realistic and ethical depiction of what an alleged mentally tough performer is actually like (Andersen, 2011).

Third, the mixed‐method approach allowed us to demonstrate how one can build an increasingly comprehensive and deeper understanding of an athlete. We started with what *type of person* Wade is (question 1). We then moved to his contextualised *goals and values* in football and how he *functions in response to demands* in his existing role at his club (question 2). Finally, we examined the subjective story he crafts that gives *meaning and purpose* to his existence (question 3). The study adds to the limited store of mixed‐method designs in the MT literature. It achieves triangulation and complementarity (Moran, Mathews, & Kirby, 2011) to create a more comprehensive, dynamic, and, at times, contrasting picture of an athlete than could have been achieved through just a quantitative or qualitative approach. In evaluating the worth of the design, and working towards a credible standard of merit (Sparkes, 2015), the study reflects a genuine attempt to express a sense of reality, sincerity, and coherence while understanding the psychological nature of a mentally tough competitor. Although this mixed‐method integrative analysis has been applied in personality psychology (Singer, 2005), there is still a tendency to see the use of qualitative description in opposition to quantitative trait and motive measurement. The research presented here is another demonstration of the great potential of blended approaches that do not argue for either/or pathways to personality analysis.

Lastly, an interesting element about the psychological portrait emerging from the data was the high level of coherence detected across the three layers. By coherence, we mean the extent to which the structure and function of Wade's personality can be shown to demonstrate a meaningful internal pattern that organises his life (Block, 1981; Murray, 1938). For instance, Wade's achievement motivation (very high C, themes of agency and achievement in strivings, commitment/legacy scripts), ability to cope (very low N, problem‐focused/borderline repressive style, redemptive self‐defining memories), and self‐sacrifice (high A, moral strivings, communal/religious narratives) denote some of the most consistent and defining themes that appear to promote his persistence in football, and his life generally. From a theoretical standpoint, the integration of such themes across the three layers of personality suggests this consistency may be a characteristic feature of people who demonstrate high levels of MT. Naturally, examining the interrelations across the three layers, and how they associate with MT, requires future empirical consideration. Taking this approach will help reveal the extent to which a full three‐layer model of personality coheres around MT or whether, as some scholars have suggested (e.g. Lin et al., 2017; Roberts & Woodman, 2015), the construct is reducible to a single trait or disposition, such as E or C. Furthermore, this mixed‐method approach will add to the emerging body of literature in personality psychology that has explored the predictive power and empirical relations of the three layers as essential and incrementally valid features of personality (e.g. Adler, Lodi‐Smith, Philippe, & Houle, 2016; Manczak, Zapata‐Gietl, & McAdams, 2014).

### An integrated conceptualisation of mental toughness

**Figure 2:**
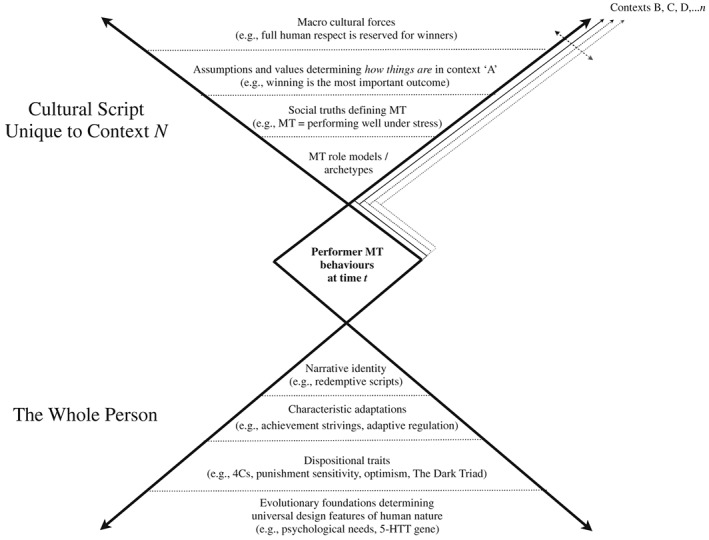
An integrated, person‐in‐context conceptualisation of mental toughness (MT).

To summarise the current research, and link it to previous literature, we offer a heuristic model for conceptualising MT that combines social, behavioural, and personality levels of analysis (Figure 2). This model showcases the person in context and illustrates the value of the McAdams framework when integrated into a broader network of scientific understanding. It also outlines the general areas covered in the present study—from initial stage of case selection through to the analysis of personality data—to examine Wade as a ‘mentally tough’ footballer. Overall, the model extends an understanding of MT in two ways. First, it endorses a view that MT can be depicted as a sporting ideal that conveys different social connotations and the ensuing promotion of certain prized (i.e. contextually valued) behaviours. Second, it illustrates that such behaviours, as public indicators of MT, can also be linked to and studied across multiple layers of personality. Several features and assumptions are central to the design of Figure 2. These key elements are now outlined and discussed in further detail. To aid this discussion, we have included some distinguishing features of MT into Figure 2, drawn from a combination of the existing MT literature (at both social and personality levels) and findings from the current research.

#### Promoting the person in context

The model is designed to represent the person in context and supports the study of mentally tough behaviours as grounded in sociocultural contexts and linked to the persons performing them. Its vertical presentation stems from a portion of Sheldon's (2004) integrated multilevel framework. Briefly, Sheldon (2004) proposed a comprehensive conceptual hierarchy that considers all the different levels, or facets, of a person—that is, the biochemical, neuronal, cognitive, personality, social, and cultural factors—that each make unique contributions to human behaviour. This hierarchy is useful for identifying all the major factors that cause any particular behaviour and recognises the cross‐level interactions among these factors at different levels of analysis. Guided by Sheldon's framework, and highlighted by the intersecting arrows, Figure 2 focuses on the interaction between social and personality dimensions for understanding mentally tough behaviour. MT behaviour is located at the centre of this interaction, which implies it is partly (i) substantiated by sociocultural forces that define what MT is in a particular context and (ii) underpinned by a performer's personality, which, in this case, is represented by McAdams' (1995, 2013) three domains. In the current study, self‐sacrificing actions and unrelenting standards symbolised desired behavioural displays of MT pertinent to case selection. Elsewhere, other behavioural themes have been linked to MT. These themes include performing well under stress (Arthur, Fitzwater, Hardy, Beattie, & Bell, 2015; Hardy et al., 2014), being adaptable in goal pursuit (Anthony, Gordon, Gucciardi, & Dawson, 2017), conforming to hyper‐masculine ideals (e.g. concealing vulnerability; Tibbert et al., 2015), responding positively to mistakes (Diment, 2014), and showing signs of moral (e.g. higher moral reasoning) and interpersonal (e.g. exhibiting prosocial norms) development (Gould, Griffes, & Carson, 2011).

#### Cultural scripts defining mental toughness

The top of Figure 2 reflects the significance social forces play in determining what MT means in a unique sporting context. Here, the influence cultural scripts have in defining what is accepted as MT, at a particular point or phase in time, is emphasised. Derived from macro levels of sporting culture (Caddick & Ryall, 2012) and linked to micro environments (e.g. organisational, subcultural; Cavallerio, Wadey, & Wagstaff, 2016; Coulter et al., 2016; Eubank et al., 2017; Tibbert et al., 2015), these scripts reflect the norms and ideals that are attached to MT in a given context, which form the subconscious/conscious indicators against which athletic behaviours (attitudes, perspectives, etc.) are judged. The model's design accounts for the possibility that different sporting contexts (i.e. B, C, D, …, *n*, or, indeed, any context that ratifies ‘MT’ in its discourse) may portray MT in different ways, subsequently influencing who is reputed as a ‘mentally tough’ role model/archetype. It also assumes a shared relationship performers have in determining what is defined as MT in a particular context. In other words, what performers do and achieve over time, and how they conduct themselves under particular conditions (e.g. stress and injury), may influence how MT comes to be understood and construed. Overall, a key feature of Figure 2 is the recognition that MT partly conveys a performer's ability to exhibit and conform to certain ways of behaving that derive from social truths determining ‘how things are’ in a particular performance context. This latitude for the social interpretation of MT is reflected in the selection procedure used in the current study to identify Wade as a mentally tough footballer.

#### The whole person

For the personal factors underpinning mentally tough behaviour, at the base of the model, room is kept for McAdams' (1995, 2013) whole person conception of personality—dispositional traits, characteristics adaptations, and narrative identity. The three layers are ordered hierarchically in Figure 2, which serves to recognise (i) that individuality develops from a universal human design (e.g. needs and genes); (ii) that in personality development, stories layer over goals and values, which in turn layer over dispositional traits; and (iii) the differential relationship personality has with culture and context, whereby social forces exert a distal effect on dispositional traits (owing to greater biological determinants), a stronger impact on characteristic adaptations, and the most profound and proximal effect on narrative identity (McAdams & Olson, 2010; McAdams & Pals, 2006). 8A vertical illustration of McAdams' framework underlines the developmental and social features distinguishing the three layers of personality. However, we recognise that a strict hierarchical design in the context of Sheldon's (2004) original framework relates more to the issue of emergence (and reduction) across different levels of scientific analysis. Whether emergence or constraint is fully applicable to the three layers of personality is a topic of scholarly debate (McAdams & Manczak, 2011). Regardless, we see merit in the logic that stories are layered over goals and motives, which are layered over dispositional traits, and, hence, feel justified with this design aspect and emphasis of Figure 2.


Using McAdams' framework, Figure 2 provides a structure for developing a greater degree of coherence in psychologically focused MT research. In particular, at its base, Figure 2 accounts for the research focused on the genetic (e.g. Golby & Sheard, 2006; Horsburgh et al., 2009; Onley et al., 2013) and universal origins (e.g. Bahmani et al., 2016) and needs (Mahoney, Gucciardi, Ntoumanis, & Mallett, 2014) shaping the underpinnings of a mentally tough personality. In line with the current opinion of personality in sport psychology (Roberts & Woodman, 2017), it also acknowledges the emphasis scholars have placed on studying MT at a trait level of analysis (e.g. Clough et al., 2002; Gucciardi et al., 2015; Guillén & Laborde, 2014; Hardy et al., 2014; Sabouri et al., 2016). The concern for traits in MT research suggests scholars have, up until now, mainly considered this foundational domain in their quest to define the mentally tough performer (Lin et al., 2017). However, as has been observed here, the solitary focus on traits neglects the opportunity to understand the whole person that, in turn, undermines the role played by other aspects of personality. For instance, at layer 2 (characteristic adaptations), these other aspects are discussed by Harmison (2011), who led a proposal to study MT behaviour using Mischel and Shoda's (1995) Cognitive Affective Processing System. Furthermore, Richards (2011) advocates the importance of self‐regulatory strategies to being mentally tough. To date, these additional perspectives of MT have largely gone unnoticed in the research literature or, in the latter area's case, been treated merely as a correlate of a MT trait rather than a core aspect of a mentally tough personality. Likewise, at layer 3 (narrative identity), a focus on traits alone disregards the types of narratives told by identified mentally tough performers (Coulter, Mallett, & Singer, 2017). It misses the prospect of enlightening the deep connections people have with sport and the internalised scripts and memories reinforcing their abilities to bounce back from setbacks, cope with failures, and persist towards their goals in the face of difficult challenges.

The presentation of McAdams' three layers in Figure 2 also helps to acknowledge the interdependency between society and personality for understanding people's mentally tough behaviour. This interdependency, and its variations across personality (McAdams & Pals, 2006), is demonstrated in the current case study (e.g. what was particularly meaningful in Wade's life story, how he reportedly copes as club captain). It is also relevant to the proposal that MT may reflect both stable and malleable aspects of personality. To make the point, we refer to Bell et al.'s (2013) recent and highly comprehensive MT development study, which involved the analysis of a 2‐year multimodel intervention programme delivered to a group of elite youth cricketers. This intervention provided a personalised psychological skills training programme that aimed to teach players to better cope with perceptions of threat during performance tasks (e.g. batting against pace and completing demanding fitness tests). It was also backed by the England and Wales Cricket Board (a UK‐based sport national governing body), delivered in a transformational manner, and underpinned by principles of systematic desensitisation (Wolpe, 1958). Compared to a control group, at the end of the 2‐year programme, the findings suggested a significant increase in player MT, which was measured via expert coaches' behaviour ratings of players performing under stress conditions in competitive matches (Hardy et al., 2014).

To explain this positive effect, McAdams' (1995, 2013) framework, and Figure 2 more broadly, can be used to hypothesise how the intervention might have influenced the players' personalities. At layer 1, the intervention's impact is debatable given the stable nature of traits from early adolescence (McAdams & Olson, 2010). However, the players in the intervention group may have been *habitually more sensitive to punishment* or possessed other dispositional traits (e.g. generalised self‐efficacy, optimism, and alexithymia; Gucciardi et al., 2015; Roberts & Woodman, 2015) that allowed them to respond favourably to threat in stressful performance conditions (Hardy et al., 2014). At layer 2, the continuous support given to the players during the intervention period may have influenced what they wanted (i.e. their motivational agenda)—in this case, *a desire to be mentally tough* as defined by the England and Wales Cricket Board (i.e. someone capable of performing well under pressure). Also, the psychological training schedule will likely have positively impacted how these players *coped in time and context* when goal achievement was under threat. Similarly, at layer 3, with ongoing exposure to an ‘inspirational vision’ of what it takes to be a world's best player, the players in the intervention group may have formed the bases of a narrative identity *around a particular idealised image of MT* and, more broadly, their involvement in an opportunistic programme focused on creating future, super‐elite cricketers. In particular, Bell et al.'s intervention occurred at a time in the players' lives when they would have begun to consider their own place in the world (‘Who am I?’), where they might be heading (‘What do I want to do?’), and what could be their lasting legacy in the game (‘What do I want to be known for?’).

Put together, the cumulative effect of dispositional traits (habitual tendencies), characteristic adaptations (recurring goals and coping strategies), and the budding formation of a convincing life story (meaning and purpose) is one possible way of making psychological sense of the intervention's resounding success. When presented like this, it is evident that traits provide a contributing, but limited, depiction of MT. A three‐layer account of personality also suggests how MT can be conceptualised in a fashion both stable and dynamic. Specifically, it implies that mentally tough individuals will have a stable component to their personalities that is grounded in genetics and early nonshared environmental experiences (Horsburgh et al., 2009; Veselka, Schermer, Petrides, & Vernon, 2009). At the same time, however, these people's personalities will also contain other aspects that are more susceptible to change (McAdams & Olson, 2010) and manifest across what they want in their lives, how they choose to cope, or what is especially meaningful to them at a particular point or phase in time. Such change may occur through exposure to a facilitative climate (e.g. Fletcher & Sarkar, 2016) that, consequently, fosters a capacity to be mentally tough (e.g. endure adversity and deepen one's commitment to achieving challenging goals). From this perspective, McAdams' framework is promising for establishing a more refined examination of the nature–nurture debate in MT research and, equally, shaping the design and emphasis of future MT intervention studies.

#### Time

The reference to time (in the centre of the model) is made to account for the potential role this dimension plays in conceptual interpretations of MT. If one takes a socially constructed view of MT, what it means and represents in any particular context has the potential to change depending on what is valued in that context at time *t*. For example, a change of coach or coaching philosophy may significantly alter the performance climate in which athletes train and perform. In doing so, what is understood by MT may shift in its meaning and, consequently, affect how certain sport behaviours and attitudes are interpreted and desired in time and space. As alluded to above, people's personalities may also change with time. For instance, traits only begin to show signs of stability from adolescence, while people's goals and values (Tibbert et al., 2015), and the meanings they attach to critical events in their lives, may change considerably (McAdams & Olson, 2010). Hence, it seems important that time be considered as an important conceptual feature in Figure 2 for understanding MT and people's abilities to exhibit mentally tough behaviours.

#### Potential significance of Figure 2

Figure 2 is a guiding outline for generating new activity and direction in MT research, especially across the social, behavioural, and personality levels of scientific analysis (Sheldon, 2004). Perhaps the most intriguing possibilities of Figure 2 are linked to McAdams' (1995, 2013) framework as a basis for conceptualising MT across three layers of personality and examining the interdependency between personality and context for understanding MT behaviour. From this perspective, a whole person approach to studying MT encourages researchers to explore the concept within each layer of personality, whereby the efficacy of particular traits (e.g. hardiness, reinforcement sensitivity, and The Dark Triad; Clough et al., 2002; Hardy et al., 2014; Onley et al., 2013), characteristic adaptations (e.g. schemas, defences, and self‐regulatory strategies; Richards, 2011), and narrative constructs (e.g., self‐defining memories; Singer & Salovey, 1993) might be examined in relation to displays of MT behaviour. It may also encourage researchers to explore the importance coherence plays across the three layers to understanding the psychology of mentally tough performers. Lastly, the interaction between personality and context is not a new idea in sport psychology, nor is it in the MT literature. However, McAdams' (1995, 2013) framework enables scholars to advance how they might study the impact social constraints have on people involved in sport. In the case of MT, it encourages greater sophistication in study designs for investigating the whole person. This investigation might involve examining how social scripts (e.g. master narratives in the family or sporting organisation) and contingencies (e.g. coaching climates), which define MT, differently influence (i) the type of individual that is considered to be mentally tough; (ii) what people want and value in sport, and how they choose to commit and cope when challenged (e.g. injury and failure); and (iii) what is especially meaningful to a performer's life in time that fosters a high level of motivation and resolve.

### Limitations

Some limitations of the current study concern the constructs used to profile Wade as a mentally tough athlete. While we have selected certain constructs that correspond to each layer of McAdams' (1995, 2013) framework, and the ensuing research questions, it is debatable that we have fully captured or satisfied what the concept of MT represents in the context of sport. By introducing various personality dimensions into the fold, we might also be accused of only adding to the confusion concerning what MT is. In considering these limitations, Richards' (2011) has said:

To avoid creating a new unwieldy multidimensional construct it may be more effective and logical to consider whether existing psychological constructs, either individually or in combination, can fulfil all the requirements that the general concept of MT was focused on achieving. (p. 294)

We make no claims to have fulfilled or achieved ‘all the requirements’ deemed necessary to define MT, which is a topic of continual debate in this area (Sorensen, Schofield, & Jarden, 2016). However, at the very least, we have acknowledged the value of drawing on existing psychological constructs for understanding MT in sport. Equally, the research was a coherent and organised effort to better understand the different types of resources (traits, adaptations, story) possessed by a mentally tough competitor. Certainly, other trait measures, measures of coping and defensive styles, and narrative instruments could be used to capture the domains of personality that we examined. We also acknowledge that several of the constructs used in the study have appeared in various forms in previous MT literature (e.g. Clough et al., 2002; Connaughton et al., 2008; Connaughton, Hanton, & Jones, 2010; Hardy et al., 2014; Kaiseler et al., 2009; Nicholls, 2011). However, from a conceptual perspective, until now, there has been no overarching framework provided to organise these constructs into a systematic depiction of a sport performer's personality, and equally, how that personality integrates with social and behavioural levels of scientific analysis (Figure 2).

## Conclusion

The current study adopted a single‐case approach to profiling the personality of an athlete who was identified as being mentally tough. Drawing on contemporary perspectives in personality psychology, this profile explored the utility of McAdams' integrative framework, which promotes a three‐domain examination of psychological individuality. The findings offered a complex insight into the nature of one person's personality, in doing so, signalling an original approach to advancing the conceptual debate on MT. The study provided an exemplar of how to study personality in sport (and in general) to gain a greater depth of knowledge and understanding of a competitor (and an individual in general) from a whole person perspective. It also encouraged MT to be examined beyond a trait level of analysis and for it to be considered, more broadly, in conjunction with other levels of scientific understanding. Finally, the study was one of the first investigations to explore the utility of McAdams' model in the context of sport and, subsequently, added support to the call for a more comprehensive and encompassing definition of personality in mainstream personality psychology.
